# Arid4b alters cell cycle and cell death dynamics during mouse embryonic stem cell differentiation

**DOI:** 10.3906/biy-2009-6

**Published:** 2021-02-09

**Authors:** Gözde GÜVEN, Nihal TERZİ ÇİZMECİOĞLU

**Affiliations:** 1 Department of Biological Sciences, Faculty of Arts and Sciences, Middle East Technical University, Ankara Turkey

**Keywords:** Embryonic stem cells, chromatin, cell cycle, cell death, endoderm differentiation

## Abstract

Cell division and death play an important role in embryonic development. Cell specialization is accompanied with slow proliferation and quiescence. Cell death is important for morphogenesis. Gene expression changes during differentiation is coordinated by lineage-specific transcription factors and chromatin factors. It is not yet fully understood how alterations in gene expression and cell cycle/death mechanisms are connected. We previously identified a chromatin protein Arid4b as a critical factor for meso/endoderm differentiation of mouse embryonic stem cells (mESCs). The differentiation defect of Arid4b-deficient mESCs might be due to misregulation of cell proliferation or death. Here, we identified a role for Arid4b in cell cycle rewiring at the onset of differentiation. Arid4b-deficient differentiating cells have less proliferative capacity and their cell cycle profile is more similar to mESC stage than the differentiating wild-type cells. We found no evidence of increased DNA damage or checkpoint activation. Our investigation of cell death mechanisms found no contribution from autophagy but revealed a slight increase in Caspase-3 activation implying early apoptosis in Arid4b-deficient differentiating cells. Taken together, our data suggest Arid4b regulates cell cycle alterations during exit from pluripotency. Future studies will be instrumental in understanding whether these changes directly contribute to Arid4b-dependent differentiation control.

## 1. Introduction

Embryonic stem cells (ESC) are pluripotent and self-renewing cells that originate from the inner cell mass (ICM) of the developing blastocyst. Owing to their limitless proliferation capacity and in vitro differentiation potential, they are widely used as a model for molecular studies of early mammalian development (Niwa, 2007; Young, 2011). The ESC state is transcriptionally maintained by pluripotency transcription factors (TFs) and chromatin factors (Masui et al., 2007). Working together, they enable stable expression of the pluripotency-related gene expression network and suppress lineage specific genes (Orkin and Hochedlinger, 2011). 

Sin3a is a multisubunit corepressor complex that contains histone deacetylases, Hdac1, and Hdac2 (Kadamb et al., 2013). Arid4b is a nonenzymatic member of the Sin3a corepressor complex that belongs to the Arid family DNA binding proteins. Along with the weak DNA-binding ability, it has chromatin reader domains that might be important for complex recruitment to chromatin (Fleischer, Yun and Ayer, 2003). In ESCs, Sin3a was previously found to physically interact with Nanog and cooccupy genes that are important for pluripotency (Saunders et al., 2017)**. **We previously identified Arid4b as a critical factor for mouse ESC differentiation towards meso/endoderm (Terzi Cizmecioglu et al., 2020). Arid4b-deficient mESCs failed to upregulate meso/endoderm specific gene expression program and exhibited altered H3K27 trimethylation and acetylation in lineage specific genes.

ESC differentiation and cell cycle are closely linked. Previous studies show that G1 cyclin CDKs directly regulate Oct4, Sox2 and Nanog by phosphorylation. Inhibition of Cyclin D directs ESCs to differentiation (Liu et al., 2017). On the other hand, in ESCs, Cyclin D is highly active because of inactivation of hyperphosphorylated Rb by CDK2-cyclin E (Pauklin and Vallier, 2013). After the exit from pluripotency and the onset of differentiation, cell cycle duration is lengthened, and G1-S checkpoint is properly established. It was also found that G1 cyclins regulate phosphorylation and the stability of the pluripotency TFs at the ESC stage (Liu et al., 2017). Previous studies also showed a role for G1-phase restricted expression of TGFβ pathway TFs Smad2 and Smad3 and meso/endoderm differentiation (Pauklin and Vallier, 2013; Soufi and Dalton, 2016)**.**

We hypothesized that the differentiation defect observed in Arid4b-deficient mESCs might be due to alterations in cell proliferation or cell death. We observed that during differentiation, arid4bΔ cells reach lower cell numbers when compared to WT cells. We set out to test whether Arid4b alters cell cycle or cell death dynamics during mESC differentiation. 

## 2. Materials and methods

### 2.1. Cell culture

#### 2.1.1. mESC growth

CJ9 (WT) and mESCs that Arid4b genes deleted by the CRISPR-Cas9 system (arid4bΔ) were obtained from the laboratory of Prof. Stuart Orkin (Boston Children Hospital). CJ9 and arid4bΔ mESCs were expanded in the high glucose DMEM (Gibco, Thermo Fisher Scientific, Waltham, MA, USA), including 15% FCS (Gibco, Thermo Fisher Scientific, Waltham, MA, USA), 1% Pen-strep (Gibco, Thermo Fisher Scientific, Waltham, MA, USA), 1% Nucleoside mix (80 mg Adenine, 24 mg thymidine, 85 mg Guanosine, 73 mg Uracil, and 73 mg cytosine), 1% Glutamax (200mM, Gibco, Thermo Fisher Scientific, Waltham, MA, USA), NEAA (nonessential amino acids) (Gibco, Thermo Fisher Scientific, Waltham, MA, USA), 10^-4^ M beta-mercaptoethanol on the irradiated mouse embryonic fibroblast (MEF) and cultured at 37°C and 5% CO2. When cells were 70%-80% confluent, cells were split by using 0.25% Trypsin-EDTA (Sigma-Aldrich Corp., St. Louis, MO, USA). Previous studies suggested that the ESCs which are grown in 2i containing defined medium more homogenously expressed transcription factors that are necessary for ESC pluripotency. We optimized the ESC maintenance in 2i+LIF containing defined medium suggested by the protocol (Mulas et al., 2019). We confirmed that WT mESCs grown in 2i+LIF conditions can be differentiated to endoderm in similar kinetics as the traditional LIF containing serum medium conditions. We also validated that mESCs in 2i+LIF medium are pluripotent and do not demonstrate expression of neuroectoderm marker Sox1 (not shown). The defined media contains 50% Neurobasal (Gibco, Thermo Fisher Scientific, Waltham, MA, USA), 50% DMEM-F12 (Gibco, Thermo Fisher Scientific, Waltham, MA, USA), 0.5% N-2 Supplement (100X) (Thermo Fisher Scientific, Waltham, MA, USA), 1% 50× B-27 Supplement (Thermo Fisher Scientific, Waltham, MA, USA), 0.5% BSA (Sigma- Aldrich Corp., St. Louis, MO, USA), 1% GlutaMAX Supplement (Gibco, Thermo Fisher Scientific, Waltham, MA, USA), 1% Penicillin-Streptomycin (10.000 U/mL) (Thermo Fisher Scientific, Waltham, MA, USA), monothioglycerol (1.5x10^-4^ M final) (Sigma- Aldrich Corp., St. Louis, MO, USA) and 4% FBS (Thermo Fisher Scientific, Waltham, MA, USA). The media was supplemented with 3 µM CHIR99021 (Selleckchem), 1 µM PD0325901 (Selleckchem), and 1% LIF (Millipore). When cells were 70%-80% confluent, cells were split by using TrypLE express enzyme (1×) (Thermo Fisher Scientific, Waltham, MA, USA).

During ESC and endoderm differentiation protocols, cell growth and viability were routinely assessed by trypan blue staining, followed with counting using Countess II automated cell counter (Thermo Fisher Scientific, Waltham, MA, USA). 

#### 2.1.2. Endoderm differentiation

When CJ9 and Arid4bΔ cells reached 70%-80% confluency, they were counted the cells with trypan blue staining and seeded in 10 cm petri dishes as 7.5 × 10^5^ cells each, how and cells were grown in serum-free differentiation medium (SFD medium) that contains 75% IMDM (Gibco, Thermo Fisher Scientific, Waltham, MA, USA), 25% Ham’s F-12 Nutrient Mix, 1% GlutaMAX Supplement (Gibco, Thermo Fisher Scientific, Waltham, MA, USA), 0.5% BSA (Sigma-Aldrich Corp., St. Louis, MO, USA), 1% 50 × B-27 supplement without RA (Gibco, Thermo Fisher, Waltham, MA, USA), 0.5% 100× N-2 supplement (Gibco, Thermo Fisher, Waltham, MA, USA), 1% Glutamax (200 mM, Gibco, Thermo Fisher Scientific, Waltham, MA, USA), 10 µl/mL ascorbic acid (Sigma-Aldrich Corp., St. Louis, MO, USA), and 3 µl/mL from diluted MTG (13 µL / 1000 µL) (Sigma-Aldrich Corp., St. Louis, MO, USA). The cells were incubated at 37°C at 5% CO_2_. On the second day of differentiation, embryoid bodies (EBs) were collected in 50 mL falcon tubes and centrifuged at 1000 rpm for 5 min at 4°C. They were resuspended in Accutase (Millipore) at 37°C water bath until become a single cell. Accutase was quenched with IMDM, and the cells were centrifuged at 1500 rpm for 5 min at 4°C. They were counted and seeded 5.0 × 10^5^ cells into each 6 cm petri dishes. SFD medium was supplied with 50 µg/mL Activin A (75 ng/mL final) (R&D; 338-AC). The EBs were collected by the same method and prepared for flow cytometry and western blot analysis on the fifth day of differentiation. Cells were collected and washed with ice-cold 0.1% BSA-PBS solution and PBS solutions three times for flow cytometry and western blot, respectively. The cells that were washed with PBS were snap-freeze with dry-ice.

### 2.2. Flow cytometry 

CJ9 and arid4bΔ mESCs were collected and washed tree times with 0.1% BSA-PBS solution (FACS buffer). CJ9 cells have been separated equally as unstained, single stained with Ki67-FITC (Thermo Fisher Scientific, Waltham, MA, USA; 11-5698-80), single stained with PI (Thermo Fisher Scientific, Waltham, MA, USA; P1304MP) and double staining. The arid4bΔ cells had only double stained sample. Fixation and permeabilization procedures were performed with fix and perm cell permeabilization kit (Thermo Fisher Scientific, Waltham, MA, USA; GAS003) as the protocol suggested. Each 5 × 10^5^ cells were incubated in Ki67 and PI stains as the protocol suggested. Flow cytometry was performed with BD Accuri C6 flow cytometer and the data was analyzed with FlowJo Software.

### 2.3. Western blot

5 × 10^5^ CJ9 and arid4bΔ cells were collected, washed, and frozen for each Western Blot samples. After thawing cell lysate on the ice, proper amount of 2× Laemmli buffer (Bio-Rad Laboratories, Inc., Hercules, California, USA) including β-mercaptoethanol (BME) was used to resuspend cell pellet. The cell lysates were boiled at 95°C for 10 min for protein denaturation. After the pellets were cooled on ice, they were centrifuged for 1 min at the highest speed and the supernatants were loaded into 12% polyacrylamide gel. After electrophoresis (PAGE), proteins were transferred to nitrocellulose membrane with Biorad Trans-blot turbo transfer system (Hercules, California, USA). The membrane was blocked with 5% skimmed milk in 1× TBS-T solution at room temperature for 1 h. Then, the primary antibody incubation was done for overnight at 4°C. Antibody information’s and dilutions were written in the Table. After primary ab incubations, the membrane was washed three times with 1×TBS-T for 10 min at RT. The HRP-conjugated secondary ab incubation was performed at RT for 1 h. The information about secondary antibodies were given in Table. After the membrane was washed three times, the visualization was performed with Biorad Clarity Western ECL and Max-ECL by using Biorad ChemiDoc MP system (Hercules, California, USA).

**Table:** Table. Antibodies used in the study.

Protein Name	Brand/Cat.no.	Dilution
Cyclin E1	CST/20808S	1:1000
γH2AX	Bethyl/a300-081a	1:2000
p-ATM (s1981)	R&D/af1655	1:1000
ATM	CST/2873T	1:1000
p-p53 (ser15)	CST/9284T	1:1000
GAPDH	CST/2118S	1:5000
Lamin B1	Proteintech/66095-1-Ig	1:3000
Anti-Rabbit IgG H&L (HRP)	Abcam/ab97051	1:5000
Anti-Mouse IgG H&L (HRP)	Abcam/ab97023	1:5000

## 3. Results

### 3.1. Arid4b loss does not change the cell cycle profile at the mESC stage 

**Figure 1:**
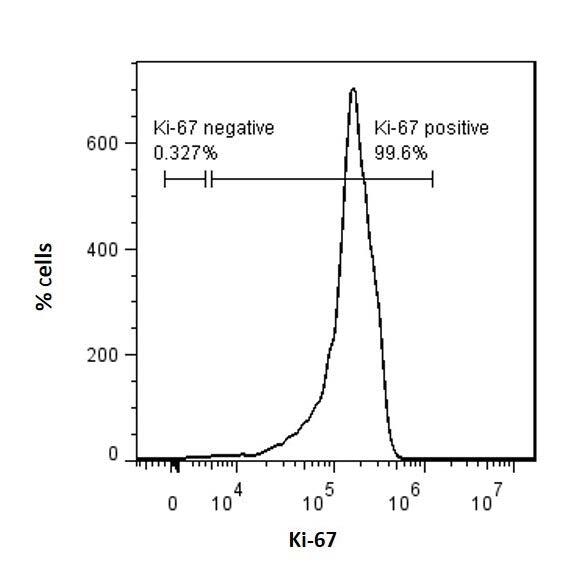

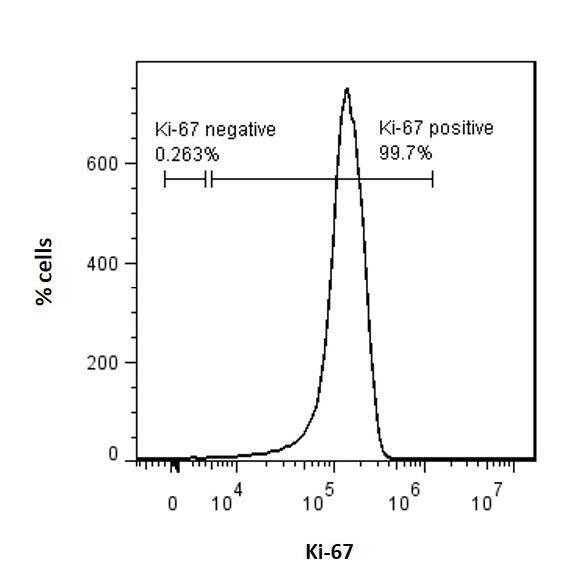

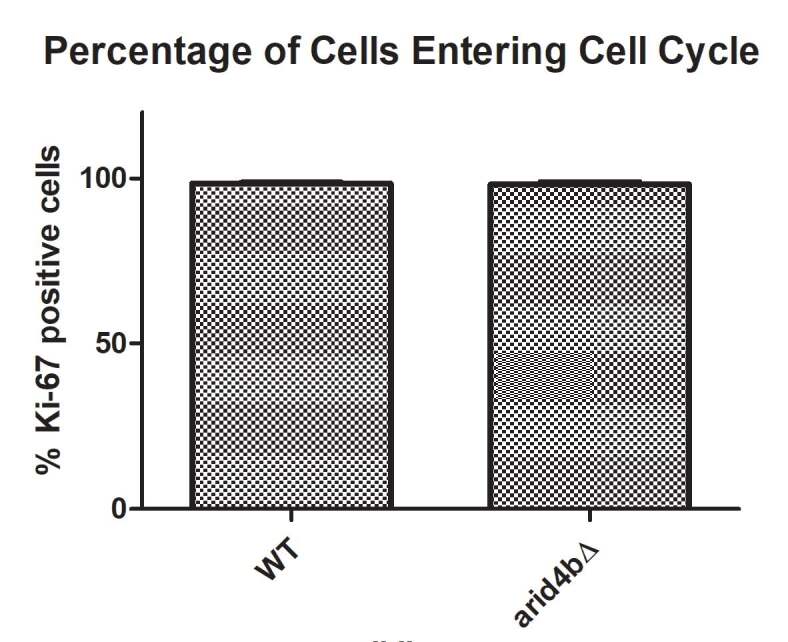

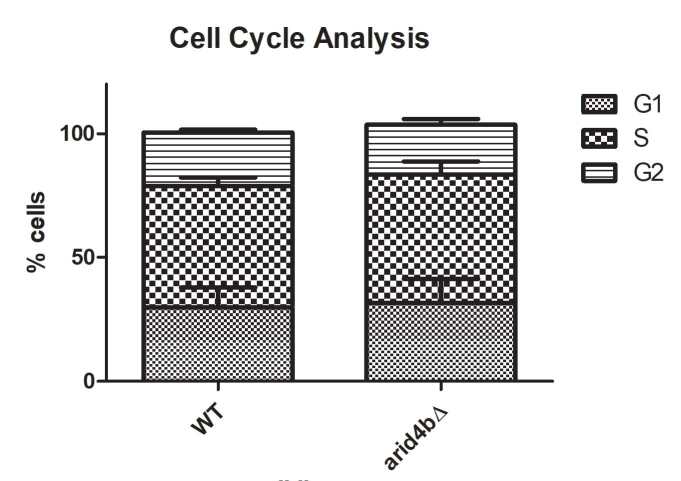

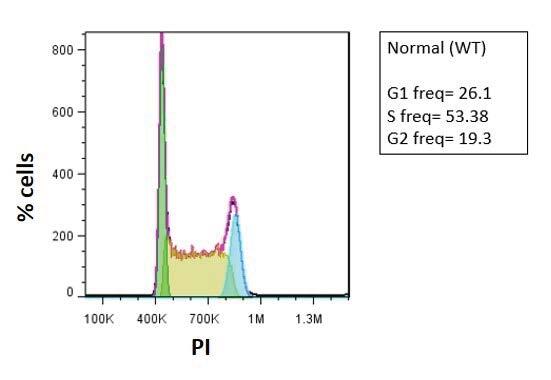

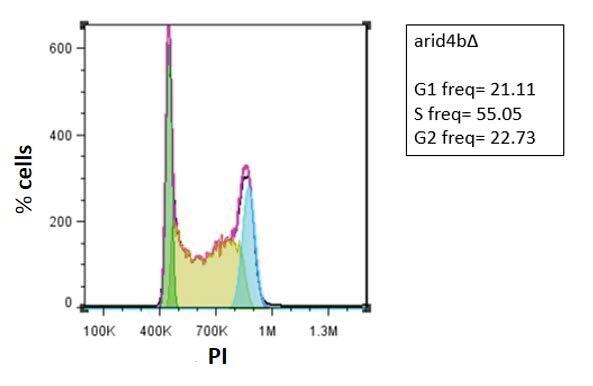
Cell cycle entry and distribution of WT and arid4bΔ mESCs. FACS histogram of a. wild type (CJ9) b. arid4bΔ mESCs determined by Ki-67-eFluor660 staining. c. Bar graph representation of Ki-67 positive WT and arid4bΔ mESCs. (Unpaired t-test: not significant) d. Cell cycle phase distribution of Ki67-positive WT and arid4bΔ mESCs. Error bars indicates standard error. (Unpaired t-test: not significant) FlowJo cell cycle analysis of e. WT and f. arid4bΔ mESCs stained with Ki67-FITC and Propidium lodide (PI). Black line represents the experimental analysis, while pink trace is cell cycle fit determined by the FlowJo software cell cycle suite. Green, yellow and blue shaded areas indicate G1, S and G2 phases, respectively.

To determine the effect of Arid4b deletion on cell cycle in mESCs, we grew wild type (WT) and arid4bΔ mESCs and labeled the cells for Ki-67 and propidium iodide (PI). Ki-67 is a protein that is highly expressed in cycling cells, whereas its expression level is decreased in G0 phase (Sun and Kaufman, 2019). Flow cytometry analysis shows that both WT and arid4bΔ ESCs are highly proliferative as depicted by extremely high percentage of Ki67-positive cells in the population (Figure 1a-1c)**.** Due to the rapid cell cycle of ESCs, they can skip cell cycle checkpoints and have longer S phase than somatic cells (Liu et al., 2019)**.** Consistent with this, PI staining of WT mESCs showed that the majority of the population is in the S-phase of cell cycle (Figure 1d-1e). arid4b∆ mESCs also exhibited similar distribution of the population across cell cycle stages (Figure 1d-1f). We conclude that the loss of Arid4b does not affect the entry into the cell cycle or the frequency of cells in various cell cycle phases at the mESC stage (not significant using unpaired t-test).

### 3.2. Arid4b loss leads to alterations of cell cycle dynamics during endoderm differentiation

**Figure 2:**
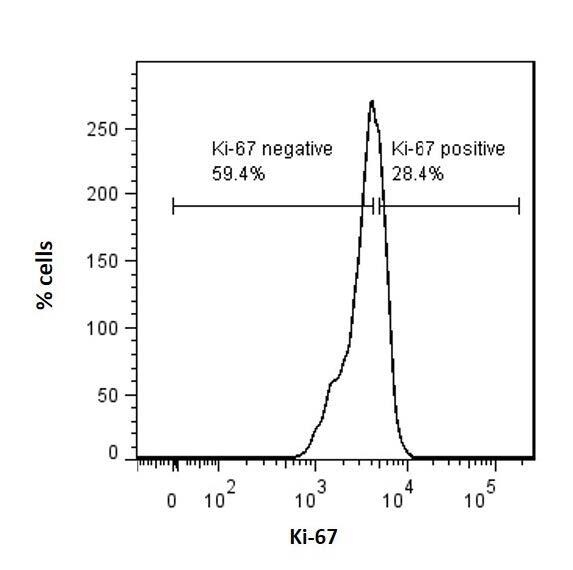

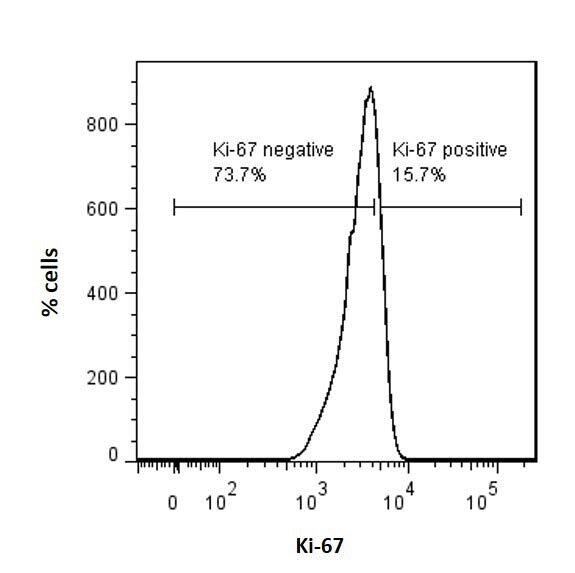

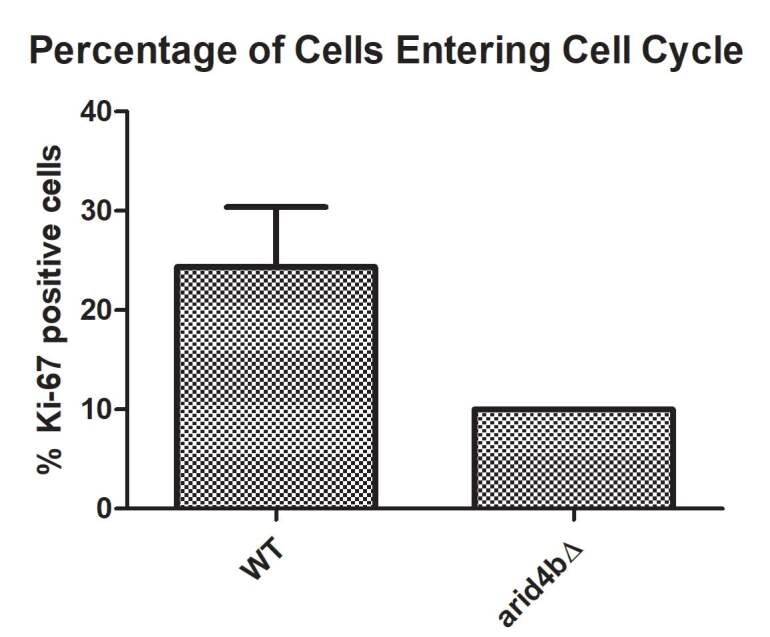

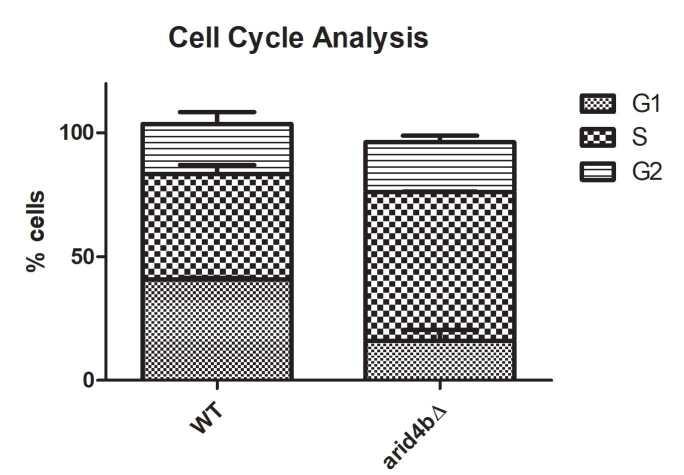

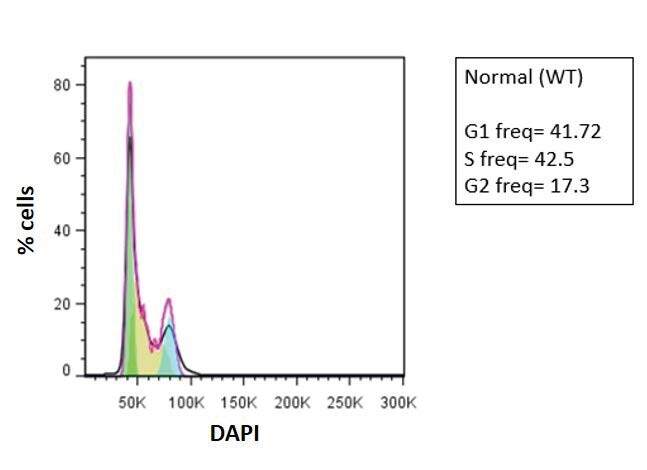

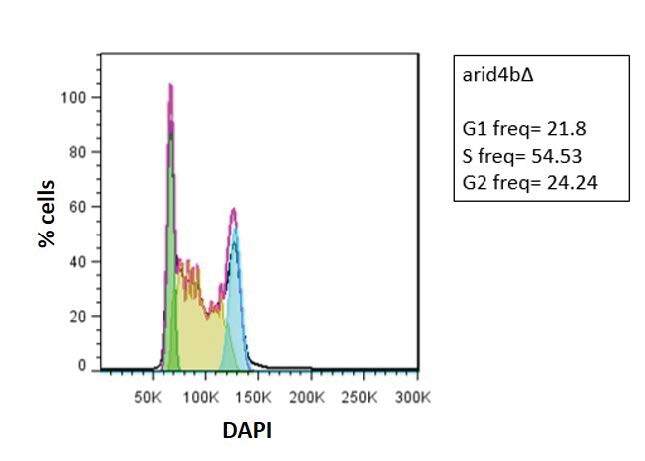
Cell cycle entry and distribution of WT and arid4bΔ mESCs. FACS histogram of a. wild type (CJ9) b. arid4bΔ endoderm directed cells determined by Ki-67-eFluor660 staining. c. Bar graph representation of Ki-67 positive WT and arid4bΔ endoderm directed cells. (Paired t-test: P < 0.05) d. Cell cycle phase distribution of Ki67-positive WT and arid4bΔ endoderm directed cells. Error bars indicates standard error. (Unpaired t-test: G1: P < 0.01, S: P < 0.01, G2: not significant) FlowJo cell cycle analysis of e. WT and f. arid4bΔ endoderm directed cells stained with Ki67-FITC and Black line represents the experimental analysis, while pink trace is cell cycle fit determined by the FlowJo software cell cycle suite. Green, yellow and blue shaded areas indicate G1, S and G2 phases, respectively.

During mESC differentiation, duration of the G1 and G2 stages increases, the checkpoints are activated, and cell cycle becomes longer (Sun and Kaufman, 2019). We, therefore, wanted to know whether Arid4b loss would alter cell cycle entry or distribution during endoderm differentiation. Using previously established protocols (Gadue et al., 2009), we directed WT and arid4bΔ mESCs towards endodermal lineage. Around day 5 of differentiation, the expression of the first endoderm specific transcription factor Foxa2 is observed (Gadue et al., 2009). Consistent with the alterations to the cell cycle, WT cells are less proliferative during differentiation when compared to the ESC stage, as depicted by Ki67 staining on day 5 of endoderm differentiation (Figure 2a). Compared to WT cells though, a lower percentage of live arid4b∆ cells were Ki67-positive (Figure 2b). Cell cycle phase distribution analysis of Ki67-positive WT cells during endoderm differentiation was consistent with previous reports (Sun and Kaufman, 2019) and exhibited an increase in the frequency of G1 phase cells (Figure 2d-2e)**.** In comparison to WT endoderm cells, a higher percentage of arid4b∆ cells were in S-phase with a concomitant decrease in the G1 phase (Figure 2d-2f). 

**Figure 3:**
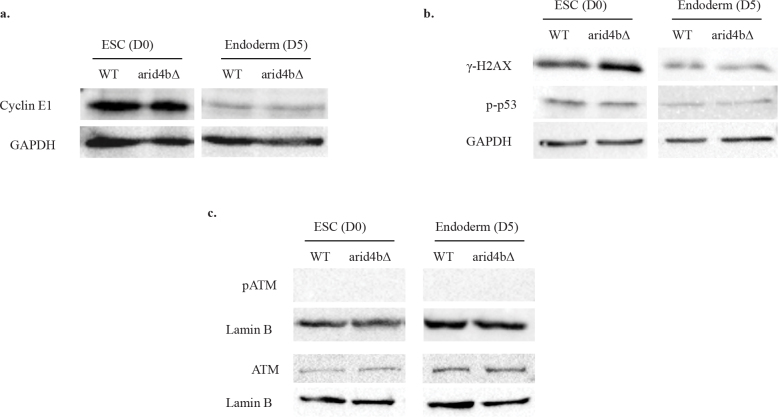
S-phase and DNA damage marker levels in WT and arid4bΔ cells. Western Blot analyses of a. Cyclin E1, b. γH2AX and p-p53 c. pATM and ATM of WT and arid4bΔ mESCs (Day0) and endoderm directed (Day5) cells. GAPDH and Lamin B were used as loading controls. The results are representative of at least three independent biological replicates.

Cyclin E1 is one of the proteins that is responsible for the passage from G1 to S phase. Cyclin E1 is maintained at a high level in rapidly dividing mESCs. On the other hand, in somatic cells, Cyclin E1 peaks at the end of the G1 phase and is slowly degraded in S phase (Liu et al., 2019)**.** As we observed higher frequency of arid4bΔ cells in S phase during endoderm differentiation, we wondered whether increased S-phase distribution of arid4b∆ endoderm cells could be explained by a difference in Cyclin E1 accumulation. Therefore, we tested the amount of Cyclin E1 level in mESCs and endoderm directed cells. Cyclin E1 level is high in both WT and arid4bΔ mESCs, consistent with high Ki67 staining at this stage (Figure 3a). As expected, Cyclin E1 level decreases after endoderm differentiation. However, no apparent difference in Cyclin E1 level was observed between arid4bΔ and WT cells. 

We also reasoned that elevated DNA damage accumulation or defective DNA repair might result in the lengthened stay of arid4b∆ cells in S-phase during endoderm differentiation. As an early marker for DNA damage, H2AX histone H2A variant gets deposited at the sites of DNA double strand breaks and is phosphorylated by ATM and ATR kinases (Kuo and Yang, 2008). ATM and ATR also phosphorylate and activate p53 for the expression of DNA damage response pathway proteins (Roos and Kaina, 2013). We examined the level of phosphorylated H2AX (γH2AX), phosphorylated ATM (pATM), and phosphorylated p53 (p-p53) proteins in mESCs and endoderm directed cells (Figure 3b-3c)**. **We did not observe any difference in the levels of these DNA damage markers between arid4b∆ and WT cells either in ESC stage or during differentiation. We conclude that the high frequency of S-phase distribution of endoderm directed arid4b∆ cells is not due to accumulation of DNA damage or defective DNA repair.

### 3.3. Arid4b loss leads to cell death during endoderm differentiation

Previously, we observed that arid4bΔ cells produce lower amounts of cells compared to WT ones during endoderm differentiation. This can be related with changes in cell cycle or/and high amounts of cell death. After we focused on the differences in cell cycle, we examined the effect of Arid4b loss on cell death mechanisms. 

Apoptosis is a programmed cell death pathway that is induced by sequential activation of caspases. Caspase 3 is one of the caspases that is activated in the early stage of the apoptosis. It is cleaved and therefore activated by Caspase 9. Caspase 3 cleavage in turn upregulates the expression of proapoptotic genes. One of the targets of Caspase 3 is PARP-1. Cleaved form of PARP-1 cannot bind DNA anymore, so DNA repair stops and genetic stability cannot be sustained anymore (Marek Los et al., 2002). To investigate this, we performed western blot analysis of cleaved Caspase 3 and PARP-1 in mESCs and endoderm directed cells**. **

**Figure 4:**
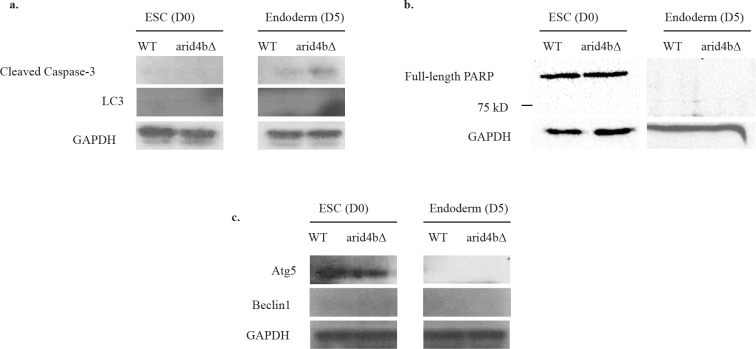
Cell death pathway activation levels in WT and arid4bΔ cells. Western Blot analyses of a. Cleaved caspase-3 and LC3, b. PARP1 and c. Atg 5 and Beclin1 in WT and arid4bΔ mESCs and endoderm directed cells. GAPDH is used as a loading control. The results are representative of at least three independent biological replicates.

In ESC stage, we did not observe any accumulation of cleaved Caspase 3 or cleaved PARP1 (~90 kD), which indicates that there is negligible if any apoptosis in WT or arid4b∆ mESCs (Figure 4a-4b). However, we found that endoderm directed arid4b∆ cells had an elevated level of cleaved Caspase 3 compared to WT, indicative of activation of apoptosis. Surprisingly, there is no cleaved PARP-1 (~90 kD) signal in either ESCs or endoderm directed cells, suggesting an early phase of apoptosis in arid4b∆ cells.

Autophagy is a cellular degradation mechanism that is activated under stress conditions such as starvation (Yu et al., 2018). We tested whether elevated autophagy response can explain the decreased proliferation of endoderm directed arid4b∆ cells. Using western blot analysis, we compared the expression of autophagy markers Beclin1, Atg5, and Lc3 in WT and arid4b∆ cells both in ESC stage as well as during endoderm differentiation. Beclin1 is one of the proteins that initiate the formation of autophagosome. Atg5 and Lc3 are found on the phagosomal vesicles and direct the autophagy (Kang et al., 2011). We did not observe accumulation of any of the tested autophagy markers in arid4b∆ cells in either ESC or endoderm stage (Figure 4a, 4c). We conclude that Arid4b loss does not alter the autophagy pathway. 

## 3. Discussion

We previously showed that arid4b∆ mESCs are defective in differentiation towards meso/endoderm (Terzi Cizmecioglu et al., 2020). There is a strong link between ESC differentiation and rewiring of ESC cell cycle (Lange and Calegari, 2010). Here, we examined the effect of Arid4b deletion on cell cycle and cell death in mESCs as well as endoderm differentiated cells. Our results reveal a role of Arid4b in regulation of these processes specifically during endoderm differentiation. As expected, we found that WT mESC population exhibits decreased Ki67 positivity as well as decrease in G1 to S transition after endoderm differentiation. Even though WT and arid4b∆ mESCs proliferate in similar rates in ESC stage, we found that the percentage of proliferating arid4bΔ endoderm directed cells is significantly lower than WT cells. Importantly, unlike WT cells, we did not observe any significant alteration of cell cycle phase distribution in arid4b∆ cells upon differentiation. Our finding shows that a high percentage of arid4bΔ cells reside in S phase during endoderm differentiation might stem from defects in the establishment of cell cycle checkpoints upon ESC differentiation, accumulation of DNA damage, or defects in DNA repair pathways. We found no evidence of increased DNA damage or checkpoint activation, suggesting Arid4b-dependent changes do not get sensed by these mechanisms. ESCs have very short gap phases (G1 and G2) compared to somatic cells, partially owing to hyperphosphorylated Rb (Lange and Calegari, 2010). We did not observe any difference in the level of G1-S transition specific Cyclin E1 between WT and arid4bΔ cells in either ESC stage or endoderm differentiation, suggesting G1 to S phase transition happens successfully in these cells. Our analyses of DNA damage and DNA repair pathway markers revealed endoderm directed arid4b∆ cells are not burdened with high level of DNA damage or a failure of DNA repair pathway. Slow, delayed, or failed DNA replication may also lead to accumulation of cells in S phase (Willis and Rhind, 2009). A deeper understanding of defective G1 and S phase rewiring of endoderm directed arid4b∆ cells requires further analysis of a complete panel of cyclins, CDKs, DNA replication, and checkpoint markers. 

We complemented cell cycle data with the effect of Arid4b loss on cell death. Cleaved form of Caspase 3 is an indicator of apoptosis and it in turn cleaves PARP1. Even though cleaved PARP1 was not observed in either ESC stage or endoderm directed cells, arid4bΔ endoderm directed cells show increased amount of cleaved Caspase 3, suggestive of early stage apoptosis (Roos and Kaina, 2013). However, no activation of authophagic response was observed in arid4b∆ mESCs or endoderm directed cells. Our results preliminarily convey a possible role of Arid4b loss in initiation of apoptosis upon ESC differentiation. Further examination of the downstream effects of Caspase 3 cleavage is required to understand how Arid4b mechanistically connects cell death with ESC differentiation.

Taken together, our data points to a role of Arid4b in regulating cell cycle alterations around the exit from pluripotency. Future studies will be instrumental in understanding whether these changes directly contribute to the meso/endodermal differentiation defect of Arid4b loss.
